# Road to sexual maturity: Behavioral event schedule from eclosion to first mating in each sex of *Drosophila melanogaster*

**DOI:** 10.1016/j.isci.2023.107502

**Published:** 2023-07-28

**Authors:** Ki-Hyeon Seong, Tadashi Uemura, Siu Kang

**Affiliations:** 1 Department of Liberal Arts and Human Development, Kanagawa University of Human Services, 1-10-1 Heiseicho, Yokosuka, Kanagawa 238-8522, Japan; 2 Graduate School of Biostudies, Kyoto University, Kyoto 606-8501, Japan; 3 Center for Living Systems Information Science, Kyoto University, Kyoto 606-8501, Japan; 4 Graduate School of Science and Engineering, Yamagata University, Yonezawa, Yamagata 992-8510, Japan; 5 Japan Agency for Medical Research and Development (AMED)-CREST, AMED, 1-7-1 Otemachi, Chiyoda-ku, Tokyo 100-0004, Japan

**Keywords:** Natural sciences, Biological sciences, Evolutionary biology

## Abstract

Animals achieve their first mating through the process of sexual maturation. This study examined the precise and detailed timing of a series of behavioral events, including wing expansion, first feeding, first excretion, and courtship, during sexual maturation from eclosion to first mating in *D. melanogaster*. We found that the time of first mating is genetically invariant and is not affected by light/dark cycle or food intake after eclosion. We also found sexual dimorphism in locomotor activity after eclosion, with females increasing locomotor activity earlier than males. In addition, we found a time rapidly changing from extremely low to high sexual activity in males post eclosion (named “drastic male courtship arousal” or DMCA). These behavioral traits leading up to the first mating could serve as clear indicators of sexual maturation and establish precisely timed developmental landmarks to explore further the mechanisms underlying the integration of behavioral and physiological sexual maturation.

## Highlights

• Genetic invariance of the duration to first mating from eclosion in *D. melanogaster*
• No dependence of post-eclosion light/dark cycle and food intake on first mating time
• Sexual dimorphism on the first phase transition of locomotor activity post eclosion
• Existence of DMCA time point after eclosion

## Graphical abstract

**Figure:**
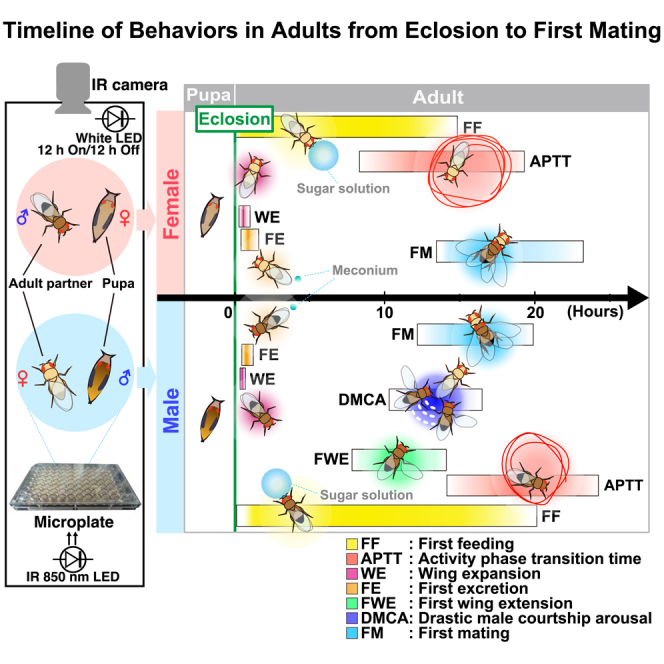

## Introduction

Comprehending the mechanisms governing sexual maturation, their intricate interdependencies, and the effects of various exogenous factors (such as nutrition, stresses, social effects, and seasonal conditions) in chronological order remains daunting. Accurately measuring the extent of sexual maturation and determining its completion is a significant challenge in many animal species due to the continuous, seamless, and prolonged development and growth.^1^ In insects, their life can be divided into two phases: the juvenile stage and the reproductive adult stage. In particular, holometabolous insects undergo drastic changes in morphology and behavior through a metamorphosis reorganizing the larval to the adult system. Additionally, acquiring sexual maturity requires a certain period of post-eclosion in many insects, thus allowing for easy measurement and quantification of the time from eclosion to sexual maturity. Therefore, holometabolous insects have a distinct advantage in understanding sexual maturation.

*Drosophila melanogaster* is a holometabolous insect and one of the most widely used model research organisms whose sexual reproductive system has been studied. *D. melanogaster* shows a stereotypical sexual courtship behavior.^2^^,^^3^^,^^4^^,^^5^^,^^6^^,^^7^^,^^8^ Recent studies have used *D. melanogaster* to elucidate the mechanisms through which the brain controls and decides the sexual reproductive process at the molecular and neuronal levels.^9^^,^^10^^,^^11^^,^^12^^,^^13^^,^^14^^,^^15^ During metamorphosis, most larval neurons survive and join with groups of new adult-specific neurons to form the adult central nervous system.^16^ The abdominal neuromeres undergo a reduction while the thoracic neuromeres experience expansion and assume the responsibility of facilitating walking and flight in the adult stage.^17^ Gross expansion and reorganization of adult neurons occur within the brain, especially in the optic lobes, the antennal lobes, and the mushroom bodies.^17^^,^^18^^,^^19^^,^^20^ Larval arbors are rapidly pruned back after pupariation, followed by adult outgrowth, target recognition, and synaptogenesis.^16^^,^^21^ During metamorphosis, glial cell numbers also increase and contribute to the reorganizing process of neural circuits.^22^^,^^23^ These processes are regulated by endocrine hormones such as ecdysone and its active derivative, 20-hydroxyecdysone.^16^^,^^21^ Through the metamorphosis, the adult flies emerge from the puparium. However, sexual immaturity persists throughout the newly eclosed adult organs, including the reproductive system and even the adult brain. The neuronal cell death occurs after eclosion and is confined predominantly to abdominal neurons.^16^ The cell death accompanies the degeneration of specific adult muscles used during ecdysis and subsequent wing expansion. The neuropeptide Bursicon induces cell death in B_AG_ neurons required for wing expansion behaviors.^24^ However, there are few studies of sexual brain maturation in the adult stage using *D. melanogaster*. Thus, well-studied knowledge of the mechanisms of the nervous system in sexual behavior has not led to an understanding of how brain maturation occurs and progresses and how they interact with the maturation of the other organs in the context of sexual maturation in adult flies.

Generally, mating comes with many costs, such as energy consumption, competition with other essential needs like feeding or sleeping, threats to predators or rivals, and physiological costs including disease, injury, or reduced lifespan.^25^^,^^26^^,^^27^^,^^28^ Therefore, mating would be driven toward a favorable energy balance for reproduction and physiologically and behaviorally low risk. However, sexual selection or sexual conflict also drives the evolution of reproductive traits.^29^ For example, seminal fluid proteins of males, such as sex peptide, regulate the sexual behavior of females in *D. melanogaster*.^28^^,^^30^ Conspecific females and males often maturate at different developmental rates (sexual bimaturism), and *D. melanogaster* shows a protogyny phenotype that females can eclose 4 h earlier than males.^31^^,^^32^^,^^33^^,^^34^ Elucidation of the detailed process and mechanism of sexual maturation could largely contribute to understanding the ecological and evolutionary aspects of the reproductive system in *D. melanogaster*.

Sexual maturity can be regarded as a spatiotemporal progression and integration of various organs, including the brain, in eclosed adult flies. The first mating is a clear indicator of completing sexual maturity in *D. melanogaster*. Therefore, elucidating the sequential flow of behavioral transitions from eclosion to first mating is significant in advancing research on the underlying organ maturation and integration processes within sexual maturity. In this study, we investigated the precise and concrete chronological schedule of several characteristic behaviors, such as wing expansion, first feeding, first excretion, and courtship behavior, exhibited by wild-type strains of male and female flies from eclosion until successful first mating in *D. melanogaster*.

## Results

### Duration until the first mating after eclosion

**Figure 1:**
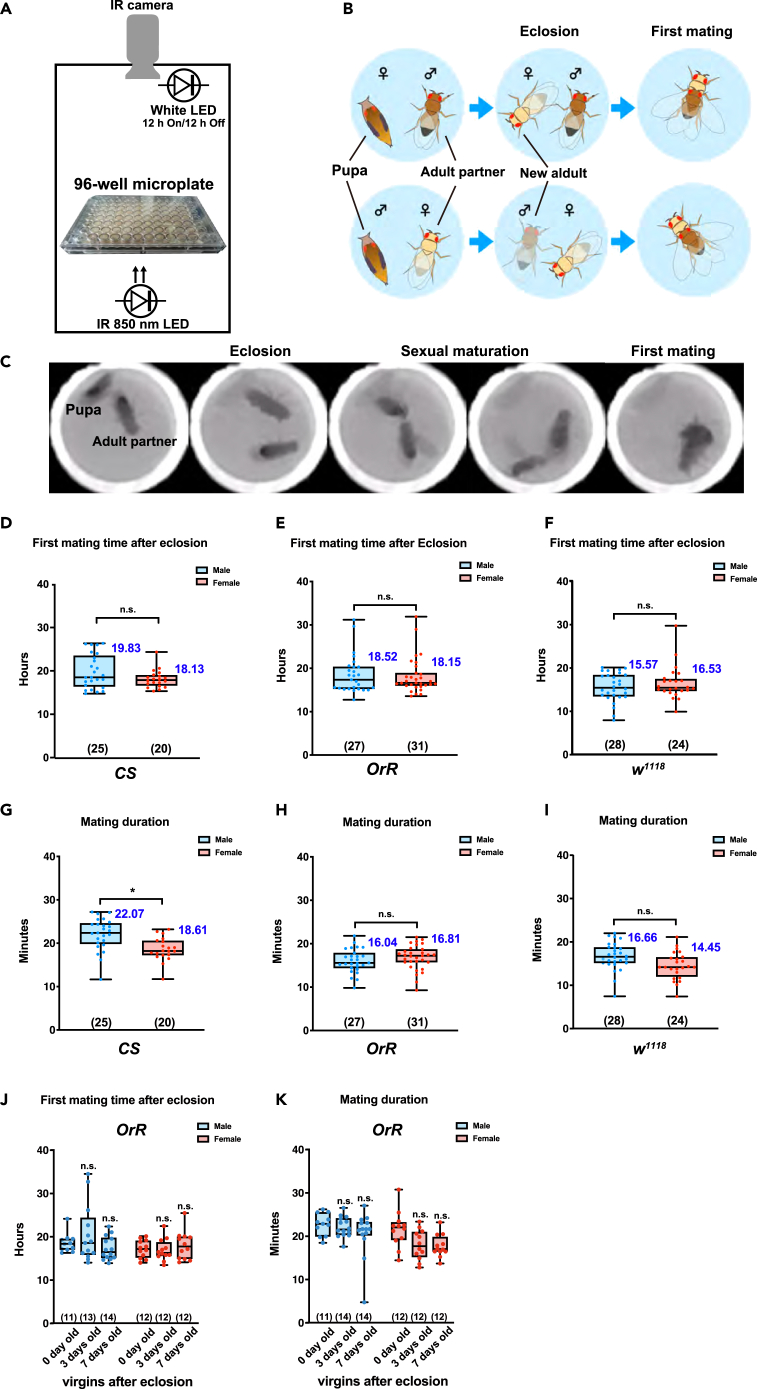
Measurement of the duration until the first mating of both sexes from eclosion in *D. melanogaster* (A and B) Schematic representations of the first mating-detection system (A) and sequences of events (B). A black pupa and a sexually mature virgin adult (five-day-old) mating partner were placed in each well of a 96-well microplate and observed until successful first mating occurred. (C) represents a timeline of representative frames captured during the observation process. (D–F) Boxplots illustrating the duration until first mating post eclosion for the three strains: (D) *Canton-S* (*CS*), (E) *Oregon-R* (*OrR*), and (F) *white*^*1118*^ (*w*^*1118*^). Whiskers indicate minima and maxima (n.s., no significant difference; Student’s unpaired *t* test). (G–I) Boxplots of the first mating duration in the (G) *CS*, (H) *OrR*, and (I) *w*^*1118*^ strains. Whiskers indicate minima and maxima (∗, p < 0.05; n.s., no significant difference; Student’s unpaired *t* test). The number of flies analyzed is indicated in parentheses in all graphs. (J and K) Boxplots depicting the (J) and mating duration (K) with the age of virgin mating partners, which were 0, 3, and 7 days old. Whiskers indicate minima and maxima (n.s., no significant difference; Student’s unpaired *t* test). The number of flies analyzed is indicated in parentheses in all graphs. See also Figure S1, Tables S1 and S2.

To determine the precise first mating time of *D. melanogaster* after eclosion, we constructed a fly behavior long-recording chamber consisting of an infrared (IR) camera, IR 850 nm LED, and white LED. The chamber can house different microplate sizes, including 96-well plates, and record the behavior of multiple individual flies under light and dark cycles (Figure 1A). A black pupa (pupal stage P14)^35^ and an adult mating partner were placed together in each well (96-well microplate) containing normal yeast-cornmeal fly medium and were recorded over 48 h (Figures 1B and 1C). Next, we visually detected time points of the eclosion and the first mating in each well (Figure 1C). We observed that almost all flies succeeded in wing expansion just after eclosion, even though the well-space was very small. We used 5-day-old virgin mating partners for the experiment, which had a high sexual demand for the opposite sex. Mature virgin partner males exhibited significantly higher courtship behavior toward newly eclosed females, while mature virgin partner females exhibited aggressive behavior, such as head-butting-like behavior, toward eclosed males. However, both newly eclosed males and females rarely conducted any sexual response in the early period after eclosion. We succeeded in detecting the precise time point of the first mating after eclosion in all strains using the 96-well microplate. The values for the first mating and mating duration in each strain are indicated in Figures 1D–1F (Table S1) and 1G–1I (Table S2), respectively. The first mating time point after eclosion did not show any significant difference between the two sexes in all three strains, yet there were differences in the first mating time between strains (Figures 1D–1F; Table S1). The duration from eclosion to first mating and the mating duration were unaffected by well size, suggesting that the confined space in the 96-well format does not disturb normal sexual maturation following eclosion (Figures S1A and S1B). We also examined the effects of mating partner age. We chose 0-, 3-, and 7-day-old virgin flies as partners for the experiment at the beginning of the observation period. Interestingly, we found no significant difference in the duration until first mating from eclosion. This result suggests that the duration until first mating from eclosion is unaffected by the sexual maturity level of the mating partner and is determined only by the individual fly’s own sexual maturation schedule (Figures 1J and 1K).

### Circadian rhythm does not affect first mating

**Figure 2:**
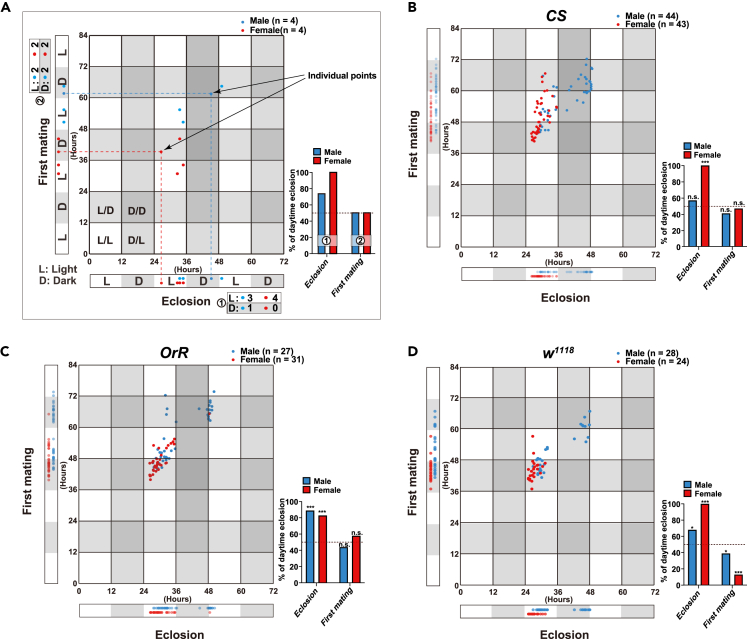
No daytime tendency for first mating in *D. melanogaster* (A) Schematic graph describing a relationship between light and dark tendency in eclosion and first mating event. The x and y axes depict the relative time of eclosion and first mating, respectively. White and gray color bands within the graph indicate light (L) and dark (D) periods on the x and y axes. The percentage of individuals undergoing eclosion and first mating during the light period was calculated by dividing the number of individuals in the light period by the total number of individuals, respectively (as depicted in the bar graph on the right side of each scatterplot). (B–D) Each scatterplot illustrates the eclosion and first mating times for the (B) *Canton-S* (*CS*), (C) *Oregon-R* (*OrR*), and (D) *white*^*1118*^ (*w*^*1118*^) strains (∗∗∗, p < 0.0001; ∗∗, p < 0.001; ∗, p < 0.05; n.s., no significant difference; Binomial test). See also Figure S2.

To assess whether the first mating is also governed by circadian rhythms, we sought to identify the time when flies succeeded in first mating under 12:12 h light and dark cycles. As expected, all strains clearly showed a tendency for eclosion to occur during the daytime in both sexes under our experimental conditions, with a more pronounced circadian daytime tendency in females (Figure 2). However, unexpectedly, the first mating did not show any daytime or nighttime tendency in *Canton-S* (*CS*) and *Oregon-R* (*OrR*) strains (Figures 2B and 2C), indicating that first mating is unaffected by circadian rhythms in these strains. Similarly, *white*^*1118*^ (*w*^*1118*^) flies showed no clear tendency for first mating during the day; however, *w*^*1118*^ females strongly preferred first mating at night (Figure 2D). We speculate that this is a result of the eclosion of *w*^*1118*^ females occurring in the early morning in a narrower range, and that the time between eclosion and first mating is slightly shorter (about 15 h after eclosion) than in the other strains (about 18 h after eclosion) (Figures 2D and 1D-1F). To further investigate the influence of the developmental stage in determining the first mating timing, we measured the time elapsed from egg-laying to first mating in the *OrR* strain (Figure S2). The eclosion event occurred primarily in the daytime and tended to avoid the nighttime in the experiment (Figures S2A–S2C). We could easily distinguish two groups (named for convenience, L (late) and E (early) groups, dotted boxes in Figures S2A and S2B), especially in male flies, when we calculated the elapsed time from egg-laying to the first mating. This gap reflected the gap in the elapsed time from egg-laying to eclosion (Figures S2A and S2B). However, we did not find a gap when the time to first mating was calculated from the eclosion time point (Figure S2D). Furthermore, the duration to first mating from eclosion had no correlation with the durations of the larval and pupal stages in both sexes (Figures S2E and S2F). These results indicate that the timing of the first mating is measured as an elapsed time after eclosion, which is independent of circadian rhythm and is independent of the previous developmental duration before eclosion.

### Gastrointestinal activity and feeding are not important for the successful first mating of both sexes

The success of normal excretion seems to reflect the normal gastrointestinal activity of the body (Figure S3). As normal gastrointestinal activity is required for effective nutrient absorption, first, we measured the first excretion (defecation of the meconium) of eclosed flies. We observed that the initial excretion was completed within approximately 2 h of eclosion, suggesting that the gastrointestinal organ of the fly may already be functioning normally within 2 h of eclosion, at least in terms of excretion function (Figures S3E–S3G). First excretion showed no daytime or nighttime tendency and did not differ between sexes in all wild-type strains (Figures S3E–S3G). The wing expansion after eclosion often occurred within approximately 30 min of eclosion (Figure S4). In most individuals, defecation of meconium was completed after wing expansion, although some individuals excreted before wing expansion (Figures S3D–S3G).

**Figure 3:**
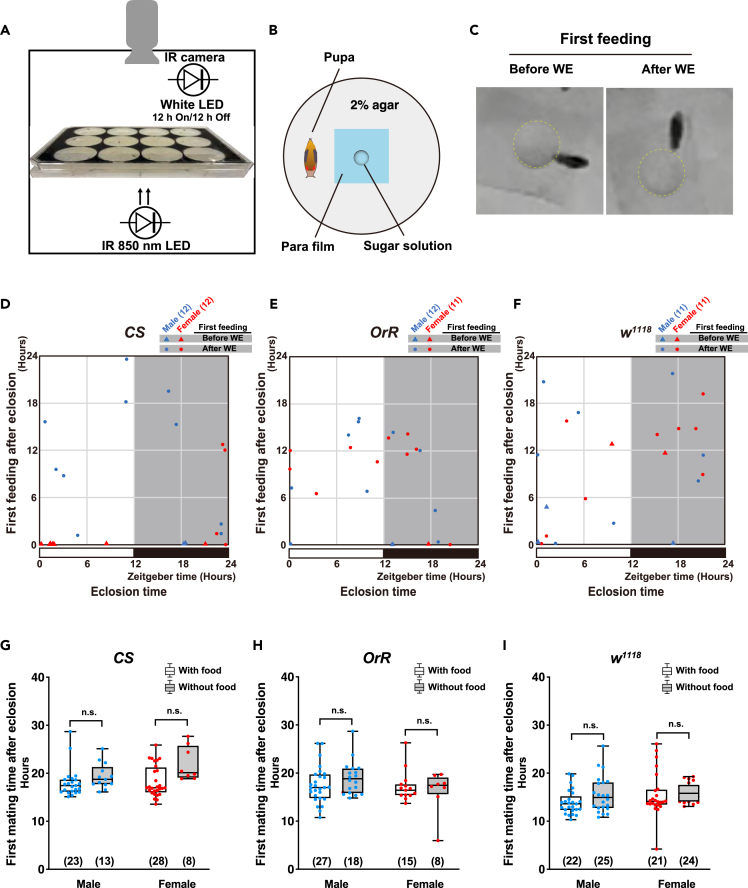
Feeding does not have significant impact on the performance of first mating in both sexes (A and B) Schematic representation of the method to detect the time of first feeding after eclosion. A black pupa was placed in each well of a 12-well microplate and observed until success of first feeding (A). A black pupa was placed in each well of a 12-well microplate containing a droplet of sugar solution on the parafilm (B) and monitored until first feeding success after eclosion. (C) Images of the first feeding before and after wing expansion (WE). Dotted lines indicate droplets of sugar solution. (D–F) Scatterplots depict the eclosion and first feeding time following eclosion in the (D) *Canton-S* (*CS*), (E) *Oregon-R* (*OrR*), and (F) *white*^*1118*^ (*w*^*1118*^) strains. (G–I) Boxplots of first mating time after eclosion with/without food in the (G) *CS*, (H) *OrR*, and (I) *w*^*1118*^ strains. Whiskers indicate minima and maxima (n.s., no significant difference; Student’s unpaired *t* test). The number of flies analyzed is indicated in parentheses on all graphs. See also Figures S3 and S4 and Tables S3–S5.

Next, we directly measured the first feeding behavior of eclosed flies as shown in Figures 3A–3C. We observed that each individual showed an extensive range of first feeding timing (Figures 3D–3F). Some individuals began their first feeding within minutes of eclosion, whereas others had their first feeding more than 10 h after eclosion (Figures 3D–3F). Unlike the relatively strict first mating time, the wide range of the first feeding time suggests that feeding may have little effect on first mating. To investigate this hypothesis further, we monitored the first mating under starvation conditions (Figures 3G–3I; Tables S3–S5). We observed that the time until first mating from eclosion was no different between the groups with or without food in all strains, indicating that feeding was not required for the performance of first mating in both sexes.

### Sexual dimorphism on the first phase transition of locomotor activity after eclosion

**Figure 4:**
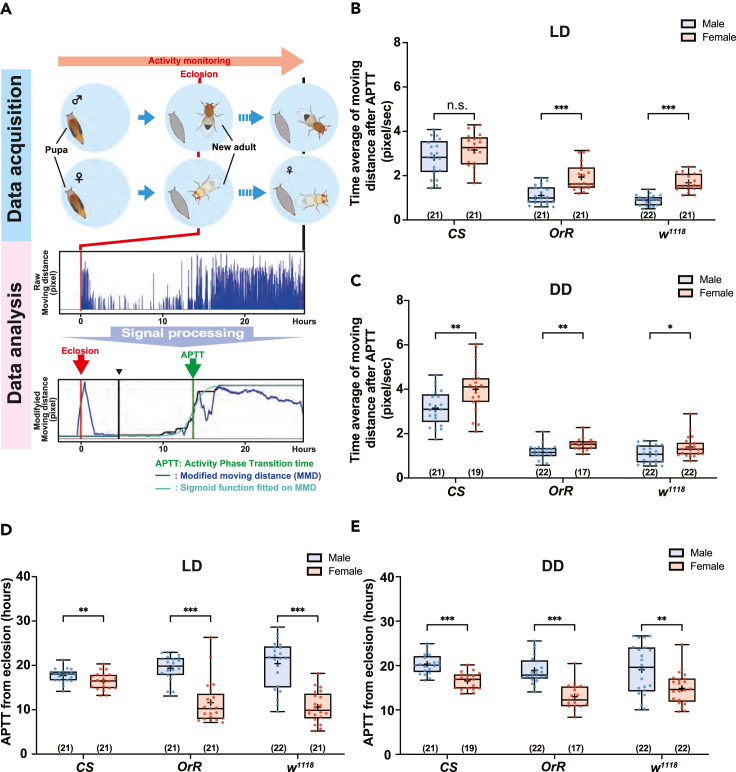
Sexual dimorphism in the time of the first activity phase transition of locomotor activity following eclosion (A) Schematic representation for monitoring and analyzing the locomotor activity following eclosion. After monitoring the activity of individual flies for two days after eclosion (upper panel), the recoded data were analyzed as following panels. Middle panels depict examples of raw activity state of fly after eclosion in the experiment. As depicted in the lower panel, the activity phase transition time (APTT) was characterized as the inflection point of the sigmoid function, derived through data processing of the raw data (see “method details”). The blue line represents the modified moving distance, and the green line corresponds to the sigmoid function fitted on the modified moving distance. The red vertical line indicates the time of eclosion, and the green vertical line indicates APTT, defined as the inflection point of the sigmoid. (B and C) Boxplots of the average direction per second after activity phase transition in the *Canton-S* (*CS*), *Oregon-R* (*OrR*), and *white*^*1118*^ (*w*^*1118*^) strains under (B) a 12:12 h light-dark (LD) cycle and (C) a dark-dark (DD) cycle conditions. (D and E) APTT of locomotor activity after eclosion in both sex of the *CS*, *OrR*, and *w*^*1118*^ strains under (D) LD and (E) DD conditions. Whiskers indicate minima and maxima (∗∗∗, p < 0.001; ∗∗, p < 0.01; ∗∗, p < 0.05; Student’s unpaired *t* test). The number of flies analyzed is indicated in parentheses in all graphs. See also Figures S5–S7 and Tables S6–S9.

To understand the relationship between the time of the first mating and the increase in behavioral activity, we next focused on a transition of the locomotor activity after eclosion. A single black pupa was placed in each well of a 24-well microplate, and the activity was monitored for 48 h under 12:12 h light:dark (LD) and dark:dark (DD) cycle conditions. Phase transitions of locomotor activity showing steep increases were analyzed. As in Figure 4A (also in and Figures S5–S7), we observed that nearly all adult flies displayed a relatively high locomotor activity immediately after eclosion. Despite the initial surge in locomotion, flies tended to quickly revert to a low-activity state that persisted for an extended period. Later, the activity gradually increased again and then shifted into a high-active state which persisted for a long period. We defined the second upward trend as a phase transition of activity and captured it by sigmoid curve fitting onto the distance that the flies moved, after some signal processing (Figures 4A and S5–S7, see also “method details”). The activity phase transition time (APTT) was characterized as the inflection point of the sigmoid function (as detailed in “Data analysis” in Figure 4A). Also, the persistent and relatively high activity after the phase transition could be estimated as the maximum value of the sigmoid function (see “method details”). Activity after APTT showed significant differences in locomotor activity between the sexes of all three strains, except for the *CS* strain in the LD condition (Figures 4B and 4C; Tables S6 and S7). The activity of CS strain under LD conditions also tended to be higher in females than males (see Figure 4B). Previous reports also noted sexual dimorphism in mature adult locomotor activity.^36^^,^^37^ We analyzed the APTT of the three strains, from a low- to high-active phase in locomotor activity in LD (Figure 4D) and DD conditions (Figure 4E). We observed that females exhibited significantly earlier APTT than males in both LD (1.3-, 7.7-, and 9.8-h difference in *CS*, *OrR*, and *w*^*1118*^ strains) and DD (3.8-, 5.9-, and 4.2-h difference in *CS*, *OrR*, and *w*^*1118*^ strains) conditions, indicating a sexual dimorphism in the duration of the phase transition to higher locomotor active phase after eclosion (Figures 4D and 4E; Tables S8 and S9). We observed sexual dimorphism in APTT but not in the first mating time. These results suggest that the sexual behavioral maturation in *D. melanogaster* is not simply synchronized with the increase in locomotor activity following eclosion.

### Drastic male courtship arousal after eclosion

**Figure 5:**
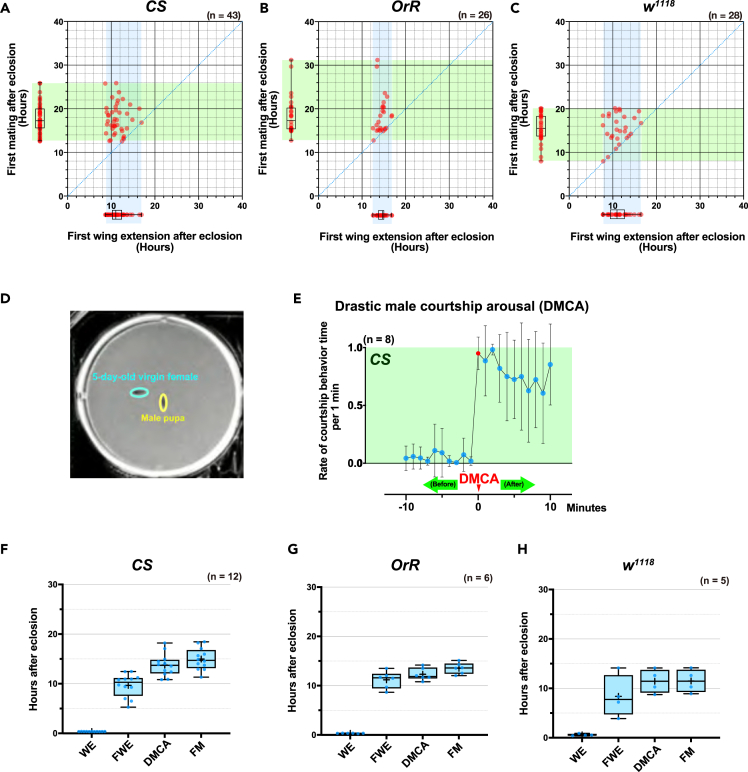
*D. melanogaster* males exhibit characteristic courtship behaviors after eclosion (A–C) Scatterplots of times of the first wing extension and first mating after eclosion in the (A) *Canton-S* (*CS*), (B) *Oregon-R* (*OrR*), and (C) *white*^*1118*^ (*w*^*1118*^) strains. Green and light blue bands within the graph indicate pointed range of minima and maxima of each axis. (D) Schematic example of the experiments. A black pupa and a sexually mature virgin adult (five-day-old) mating partner were placed in each well of a 6-well microplate and observed until successful first mating occurred. (E) Change in courtship activity rate for +/− 10 min of the drastic male courtship arousal (DMCA) point. In this paper, we define the DMCA point as the point of heightened activity at the first onset of which copulation activity increases abruptly from a near-inactive state and persists for at least 10 min. The graph illustrates fluctuations in courtship activity over a 10-min interval preceding and following the DMCA point; courtship activity on the y axis is quantified as the ratio of the duration of copulation behavior per minute (ranging from 0 to 1.0). (F–H) Boxplots depicting initial behaviors, including wing expansion (WE), first wing extension (FWE), drastic male courtship arousal (DMCA), and first mating (FM) in the (F) *CS*, (G) *OrR*, and (H) *w*^*1118*^ strains. The number of flies analyzed is specified in parentheses in all graphs. See also Figure S8.

The mating event only succeeds after an agreement between both the male and the female fly. Therefore, the first mating might also be greatly influenced by the sexual motivation of the partner. The wing extension behavior primarily reflects the internal sexual motivation of males to sexual stimuli and seems to be independent of the motivation of their female partners.^4^^,^^5^^,^^38^ Accordingly, we attempted to detect the first occurrence of wing extension behavior in eclosed male flies to comprehend the maturation of their internal sexual motivation. We detected the first mating and first wing extension in males using the same method shown in Figures 1A–1C. As expected, the variation in time of the first wing extension was narrower than that between the first mating events in all strains (Figures 5A–5C). Some males had a successful first mating at about the same time as the first wing extension, indicating that males might already be capable of mating at the time that they show wing extension behavior (Figures 5A–5C). Next, we tried to observe more clearly the male behavior just after eclosion in a wider space using a 12-well microplate (Figure 5D). Eclosed males showed a very stationary state for several hours. However, after that, we observed that males shifted to a hyperactive mode for courtship behavior (Figures 5E and S8). We were particularly interested in such rapid increases in male courtship activity that appeared for the first time after eclosion. We focused on the hyperactive modes in which courtship activation status was seen in a continuous way for at least 10 min, after a period of no courtship behavior (see the definition of courtship activation status in STAR Methods). We visually determined the initial time point of each hyperactive mode from the video, and designated it as “drastic male courtship arousal (DMCA)” (Figure 5E). We observed that the first mating for adult males occurred shortly following the DMCA in all strains (13.7, 12.3, and 11.4 h post eclosion on average for DMCA in the *CS*, *OrR*, and *w*^*1118*^ strains, respectively; 15.0, 13.5, and 11.5 h on average for the first mating, respectively), indicating that the DMCA serves as a significant behavioral turning point for males (Figures 5F–5H).

## Discussion

**Figure 6:**
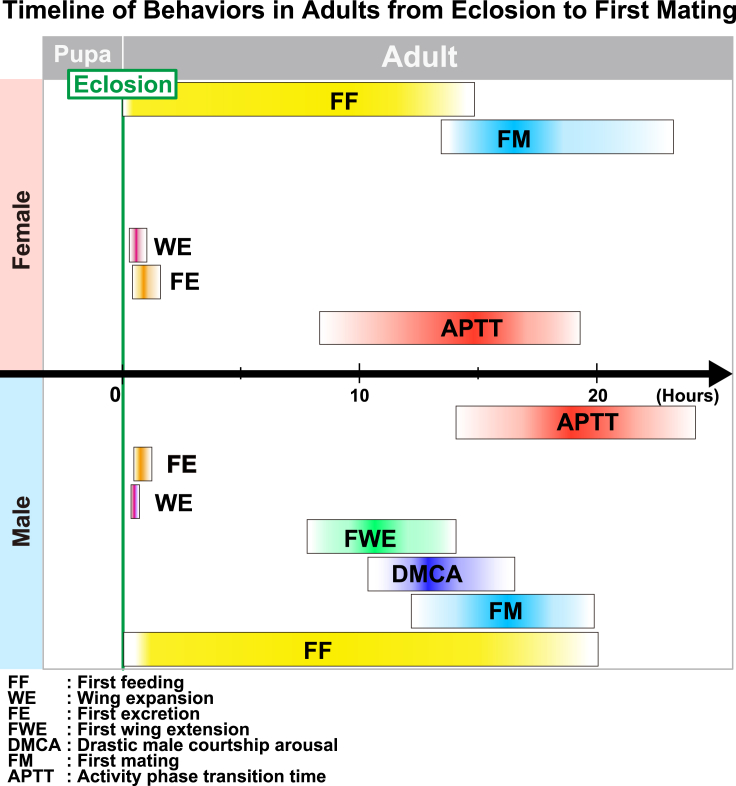
Schematic schedule of behavioral events during sexual maturation from the eclosion to the first mating in each sex This schematic represents the integrated schedule of events of three strains (*CS*, *OrR*, and *w*^*1118*^) from the eclosion to first mating, as depicted in Figure S9. The boxes depict the range of minimum and maximum values, and the darkest color and its gradations indicate the mean and distribution of each event. The following events are represented by their respective colors: yellow represents first feeding (FF); gold represents wing expansion (WE); magenta represents first excretion (FE); green represents first wing extension (FWE); blue represents drastic male courtship arousal (DMCA); light blue represents first mating (FM); and red represents activity phase transition time (APTT).

In this report, we have determined the precisely timed first mating and several characteristic behaviors after eclosion in both sexes of three different strains. The schematic timeline of behaviors during adult sexual maturation is depicted in Figure 6 (also see Figure S9).

We found that it takes a certain fixed duration for first mating after eclosion in both sexes of wild-type strains, suggesting that this process is tightly controlled genetically. We observed that the circadian rhythm was not involved in the first mating event, although the eclosion event is highly controlled by the circadian clock.^39^^,^^40^^,^^41^ It has been previously also reported that fully aged, 5- and 9-day-old virgin flies display a robust circadian rhythm in the mating activity, but 3-day-old females do not show the mating activity rhythm.^42^^,^^43^ Our results indicate that the first mating is determined by the elapsed time after eclosion in both sexes.

Generally, nutritional conditions directly affect the reproductive performance of animals, including *D. melanogaster*.^44^^,^^45^^,^^46^^,^^47^^,^^48^^,^^49^ Insulin signaling contributes to the sexual receptivity of fruit flies.^50^^,^^51^ Hence, we investigated the relationship between gastrointestinal activity, first feeding, and first mating after eclosion. We used sugar for food source in this experiment. It is noteworthy that *D. melanogaster* displays diverse responses to volatiles emitted by distinct food sources. In general, matured flies tend to consume more in the case of high carbohydrate food and, in contrast, yeast-rich diets have a pronounced suppressive effect on feeding.^52^^,^^53^^,^^54^ Therefore, our experiment may vary if a more attractive food source were employed. Nevertheless, our observations suggest that the drive for appetite in newly emerged flies may not be as intense as in hungry mature adults. Additionally, the duration until first mating from eclosion remained consistent regardless of the presence or absence of regular *Drosophila* food (yeast/glucose/cornmeal diet) during the adult stage for both males and females. These results suggest that sexual maturation after eclosion seems to progress independent of external nutrient intake.

Previously, it was reported that 20% of just-eclosed teneral females mated within 1 h.^55^ However, such forced mating to teneral females by mature males has rarely been observed in our experiments. One reason might be the difference between the experimental conditions of the female in our study and those in previous reports. We used pre-eclosed pupal females for the experiment, whereas the earlier study used teneral females collected with no anesthesia soon after eclosion.^55^

A previous report indicated that courtship depends on the internal state of the male.^56^ Our study found that males exhibit a notable turning point in sexual behavior characterized by a surge of active courtship behavior toward virgin females (termed DMCA) and first mating often coupled with DMCA. The maturation of the sensory system to sense females is important for male courtship response.^7^^,^^38^^,^^57^^,^^58^ The male-specific *fru*/*dsx* co-expressing P1 cluster is deeply connected to the decision process of male courtship.^59^^,^^60^^,^^61^^,^^62^ In addition to establishing such a dedicated neural circuitry, there is also the possibility that other factors, such as the maturation of reproductive organs, the physiological environment, or extrinsic environmental stimuli, may be involved in DMCA. Therefore, the DMCA, as a clear indicator of male sexual maturation, could contribute to elucidating the mechanism of sexual maturation by underlining the relationship between several factors.

In *D. melanogaster*, there is apparent sexual dimorphism in locomotor activity.^36^ Previous reports indicate that the sexual dimorphism in locomotor activity is genetically controlled, and the pars intercerebralis neurons directly regulate the sexual dimorphic behavior.^36^^,^^63^ We confirmed that the sexual dimorphism of locomotor activity was already exhibited after entering the active phase after eclosion. We also found that females are earlier than males to change the motivation to the active phase of locomotor activity after eclosion. Previously, we reported that females eclose 4 h faster than males in *D. melanogaster*.^32^^,^^33^ Sex differences in the duration of eclosion and maturation and locomotor activity produce a spatiotemporal gap between the sexes in siblings. Therefore, these results could be a compelling example to explain that protogyny (where females emerge earlier than males) has ecological implications for avoiding inbreeding.

In this study, we focused on the behavioral aspect of adult maturation in each sex of fruit flies. However, it is also known that the reproductive capacity is very low in very young flies just after eclosion, even if the fly has already acquired the ability to mate,^64^^,^^65^^,^^66^ suggesting that behavioral sexual maturation may occur before physiological sexual maturation. It is worth considering the benefits and costs of mating and reproduction in the physiologically immature adult stage. So far, the driving forces for the evolution of the maturation balance between behavior and physiology are not known; yet, there could be a multitude of factors influencing the optimization of the behavioral and physiological balance.^65^ Our research will help to further our understanding of the link between behavioral and physiological maturation in *D. melanogaster*.

### Limitations of the study

In this study, by putting a pupa and an adult virgin mating partner in the same well and recording video for long periods, we could precisely detect the duration of first mating from eclosion in *D. melanogaster* and obtain the new findings described above. However, it is essential to note that this analysis has presented formidable challenges and required a substantial investment of time, primarily due to the reliance on visual observations for detecting all behavioral patterns. Consequently, improving efficiency by automating detection is a major challenge for using this method. Moreover, it is imperative to acknowledge the limitations of this study, which focused solely on the behavioral interactions between a single male and a single female, thus precluding an examination of how the sexual maturation mechanism is influenced in the presence of multiple individuals. Therefore, future investigations should aim to develop methods that can analyze the effects of various environmental and physiological factors on the behavioral progression from eclosion to first mating, encompassing not only pairs but also larger groups of individuals.

## STAR★Methods

### Key resources table

**Table:** 

REAGENT or RESOURCE	SOURCE	IDENTIFIER
**Experimental models: Organisms/strains**		

*Oregon-R*	Kept in lab stock	
*Canton-S*	Kept in lab stock	
*w*^*1118*^	Kept in lab stock	

**Software and algorithms**		

Prism v. 8.4.3	GraphPad Software, LLC.	https://www.graphpad.com

### Resource availability

#### Lead contact

Further information and requests for resources should be directed to and will be fulfilled by the lead contact, Ki-Hyeon Seong (seong-4rw@kuhs.ac.jp).

#### Materials availability

This study did not generate any new unique reagents.

### Experimental model and subject details

#### Fly strains and maintenance

In this study, we examined the timing of first mating after eclosion in males and females of three different strains: *Oregon-R* (*OrR*), *Canton-S* (*CS*), and *white*^*1118*^ (*w*^*1118*^). While *w*^*1118*^ is utilized as a laboratory "wild-type" strain in *Drosophila* research, including studies of the circadian clock,^67^^,^^68^^,^^69^^,^^70^^,^^71^ it is known to exhibit visual defects^72^^,^^73^^,^^74^ and various behavioral abnormalities, including altered locomotor activity,^75^ memory,^76^^,^^77^ and courtship behavior.^78^^,^^79^ All strains were cultured in standard conditions: on standard fly medium in a plastic vial at 25°C.^80^ We recorded flies at the black pupal stage (stage P14) to observe the behavior of flies just from the eclosion. We also used 5-day-old virgin females or males as mating partners for each fly in the study.

### Method details

#### Measurement of the duration until first mating after eclosion

We designed an apparatus to record fly behavior for a long duration with the day/night cycle as described in the main text and Figure 1A. The microplate can be set at a fixed position in the chamber and was illuminated using an IR 850 nm LED placed beneath and was monitored by the IR camera placed at the top of the chamber. Recording and illumination for the day/night cycle were controlled by using the Raspberry Pi 3 Model B (https://www.raspberrypi.org/). The microplates were filled with a normal fly medium (about the half volume of the well). Then, a black pupa (stage P14) was placed in each well and sealed with Titer stick film (Watson, Tokyo, Japan). The film on each well was cut using a cutter in a cross shape, and an adult mating partner fly was placed into each well. Using the monitoring system, we recorded the behavior of eclosed flies from eclosion to first mating. Time points of eclosion and first mating were visually determined from the video.

To measure the time elapsed from egg laying to first mating, we collected the embryos in a narrow time range (30 min) and put each embryo into a well of a 96-well microplate containing fly food. Then, the pupariation time points were measured using DIAMonDS.^32^ After pupariation, black pupae (stage P14) and 5-day-old virgin mating partners were paired together in single wells of a 96-well microplate, and then monitored to detect eclosion and the first mating of each individual.

#### Detection of DMCA

To detect DMCA, the 6-well microplate was filled with a normal fly medium. A black male pupa (stage P14) was placed in each well and add a maturated virgin female (5-days old). Then, the microplate was recorded by the monitoring system and DMCA was visually determined from the video. DMCA was defined as the initial time point after eclosion when courtship response occurred and continued for at least 10 minutes, following a period of no courtship behavior. Courtship activation status is distinguished by the elicitation of one or more behaviors, such as orienting, following, tapping, wing extension, licking, abdomen bending, attempted copulation, and copulation, within a one-minute interval.

#### Detection of individual activity phase transition time (APTT) after eclosion

To measure the individual activity of adult flies just from eclosion, the black pupae were placed in the well of a 24-well microplate with normal medium. The plate was then placed on the recording apparatus described in Figure 1A. Video recording was continued for 48 hours.

To process the image, the movies obtained by continuous monitoring were transferred to sequential time-lapse images. The consecutive image frames were also divided into individual wells. All the animals were independently extracted in a well manually created as square shape images with 68 x 68 pixels. A moving animal body was detected as a foreground object separated from the background by an image processing technique called MOG which employed a mixture Gaussian distribution1 (Figure S5A). Grayscale transformation and binarization by thresholding were also applied (Figure S5B). In addition, closing- and opening-operation which are types of morphological filtering were performed for filling object and noise reduction, respectively2 (Figures S5C and S5D). An object with sufficiently large areas (>30 pixels) in a frame was determined as a moving animal body.

The trajectory of the animal body was calculated to estimate the behavioral history of an individual moving displacement. Instantaneous displacements of a fly were obtained as a moving distance of gravity center of the region of interest (ROI) between pre- and post-images. The total movement of the animal body was characterized as a sequence of instantaneous displacements.

Eclosion times of all animals were automatically detected as the first frame showing non-zero activity and additional manual confirmation. To eliminate tentative active state related with eclosion event, activity of eclosion just after 2 hours was replaced with zero. Smoothing by moving average was performed on the time series of fly displacement for 30 hours after eclosion offset (Figure S5E). To capture the distinct activity elevation timing, the time series after the smoothing was transformed as follows:

(Equation 1) $$y_{n}^{\mathrm{mod}}=\max\left(y_{1,}\;...,y_{n}\right)$$

Here, n is a frame number, $y_{n}^{\mathrm{mod}}$ is modified signal of n-th frame and y_i_ is original signal in i-th frame.

Finally, curve fitting by sigmoid function was performed on the modified signal. The rise timing of fitted sigmoid was defined as the timing of fly activity elevation after eclosion.

#### First feeding and excretion times after eclosion

We poured 2% agar into a 12-well microplate, placed a parafilm sheet (about 7 × 7 mm^2^) on the agar, and placed one crystal of sucrose on the center of the parafilm as shown in Figure 3B. After placing the sucrose crystal on the parafilm sheet, it was melted by water vapor and became a drop of saturated sucrose solution. Then, a black pupa (stage P14) was placed at the side of the agar plate and monitored the plate using the recording system described in Figure 1A for 2 days. We defined the time at which the proboscis extension reaction occurs as the index of feeding reaction, and visually measured the time of first feeding after eclosion from the video. We also visually determined the time of the first excretion after eclosion by detecting the meconium on the plate from the same video.

### Quantification and statistical analysis

Data were analyzed, and graphs were plotted using the GraphPad Prism v. 8.4.3 software (GraphPad Software, San Diego, CA, USA). A student’s unpaired, two-tailed *t*-test was performed to compare differences between groups in each experiment (∗∗∗p < 0.001; ∗∗p < 0.01; ∗p < 0.05; n.s., no significance). In order to quantify grouping factors on behavioral schedule, we also performed two-way ANOVA for sex-strain and sex-food before the direct pairwise comparisons by *t*-test. While the duration until first mating after eclosion did not show significant interaction between sex and strain (Table S1, see also Figures 1D–1F), mating duration exhibited significant interaction between them (Table S2, see also Figures 1G–1I). There were no significant interactions between sex and strain in the APTT and moving distance after the APTT in both of LD/DD (Tables S6–S9, see also Figures 4B–4E). When we tested sex-food interactions, no significant interactions were found in the duration to first mating from eclosion for all strains (Tables S3–S5, see also Figures 3G–3I).

We also performed the binomial test to characterize the light-dark (LD) tendency in eclosion and first mating. A significance level of difference from neutral (50%) was labeled as same as the *t*-test described above (Figures 2B–2D and S2C).

## Data Availability

• All data reported in this paper will be shared by the lead contact upon request. • This study did not generate/analyze any dataset/code. • Any additional information required to reanalyze the data reported in this paper is available from the lead contact upon request.

## Supplemental information

Documents S1. Figures S1–S9 and Tables S1–S9

## References

[1] Picut C.A., Remick A.K. (2017). Impact of Age on the Male Reproductive System from the Pathologist's Perspective. Toxicol. Pathol..

[2] Hall J.C. (2002). Courtship lite: a personal history of reproductive behavioral neurogenetics in *Drosophila*. J. Neurogenet..

[3] Greenspan R.J., Ferveur J.F. (2000). Courtship in *Drosophila*. Annu. Rev. Genet..

[4] Hall J.C. (1994). The mating of a fly. Science.

[5] Spieth H.T. (1974). Courtship behavior in *Drosophila*. Annu. Rev. Entomol..

[6] Ferveur J.F. (2010). *Drosophila* female courtship and mating behaviors: sensory signals, genes, neural structures and evolution. Curr. Opin. Neurobiol..

[7] Amrein H. (2004). Pheromone perception and behavior in *Drosophila*. Curr. Opin. Neurobiol..

[8] Yamamoto D., Jallon J.M., Komatsu A. (1997). Genetic dissection of sexual behavior in *Drosophila melanogaster*. Annu. Rev. Entomol..

[9] Aranha M.M., Vasconcelos M.L. (2018). Deciphering *Drosophila* female innate behaviors. Curr. Opin. Neurobiol..

[10] Ellendersen B.E., von Philipsborn A.C. (2017). Neuronal modulation of *D. melanogaster* sexual behaviour. Curr. Opin. Insect Sci..

[11] Pavlou H.J., Goodwin S.F. (2013). Courtship behavior in *Drosophila melanogaster*: towards a 'courtship connectome'. Curr. Opin. Neurobiol..

[12] Auer T.O., Benton R. (2016). Sexual circuitry in *Drosophila*. Curr. Opin. Neurobiol..

[13] Anreiter I., Biergans S.D., Sokolowski M.B. (2019). Epigenetic regulation of behavior in *Drosophila melanogaster*. Curr. Opin. Behav. Sci..

[14] Sato K., Goto J., Yamamoto D. (2019). Sex Mysteries of the Fly Courtship Master Regulator Fruitless. Front. Behav. Neurosci..

[15] Lee S.S., Wu M.N. (2020). Neural circuit mechanisms encoding motivational states in *Drosophila*. Curr. Opin. Neurobiol..

[16] Truman J., Taylor B., Awad T. (1993). The Development of *Drosophila Melanogaster*.

[17] Technau G.M. (2007). Fiber number in the mushroom bodies of adult *Drosophila melanogaster* depends on age, sex and experience. J. Neurogenet..

[18] Jefferis G.S.X.E., Marin E.C., Watts R.J., Luo L. (2002). Development of neuronal connectivity in *Drosophila* antennal lobes and mushroom bodies. Curr. Opin. Neurobiol..

[19] Gibbs S.M., Truman J.W. (1998). Nitric oxide and cyclic GMP regulate retinal patterning in the optic lobe of *Drosophila*. Neuron.

[20] Armstrong J.D., de Belle J.S., Wang Z., Kaiser K. (1998). Metamorphosis of the Mushroom Bodies; Large-Scale Rearrangements of the Neural Substrates for Associative Learning and Memory in *Drosophila*. Learn. Mem..

[21] Truman J.W., Riddiford L.M. (2023). *Drosophila* postembryonic nervous system development: a model for the endocrine control of development. Genetics.

[22] Awasaki T., Tatsumi R., Takahashi K., Arai K., Nakanishi Y., Ueda R., Ito K. (2006). Essential role of the apoptotic cell engulfment genes *draper* and *ced-6* in programmed axon pruning during *Drosophila* metamorphosis. Neuron.

[23] Hartenstein V. (2011). Morphological diversity and development of glia in *Drosophila*. Glia.

[24] Peabody N.C., Diao F., Luan H., Wang H., Dewey E.M., Honegger H.W., White B.H. (2008). Bursicon functions within the *Drosophila* CNS to modulate wing expansion behavior, hormone secretion, and cell death. J. Neurosci..

[25] Daly M. (1978). The Cost of Mating. Am. Nat..

[26] Emery Thompson M., Georgiev A.V. (2014). The High Price of Success: Costs of Mating Effort in Male Primates. Int. J. Primatol..

[27] Karigo T., Deutsch D. (2022). Flexibility of neural circuits regulating mating behaviors in mice and flies. Front. Neural Circuits.

[28] Wigby S., Chapman T. (2005). Sex peptide causes mating costs in female *Drosophila melanogaster*. Curr. Biol..

[29] Shuker D.M., Shuker D., Simmons L. (2014). The Evolution of Insect Mating Systems.

[30] Hollis B., Koppik M., Wensing K.U., Ruhmann H., Genzoni E., Erkosar B., Kawecki T.J., Fricke C., Keller L. (2019). Sexual conflict drives male manipulation of female postmating responses in *Drosophila melanogaster*. Proc. Natl. Acad. Sci. USA.

[31] Teder T., Kaasik A., Taits K., Tammaru T. (2021). Why do males emerge before females? Sexual size dimorphism drives sexual bimaturism in insects. Biol. Rev. Camb. Philos. Soc..

[32] Seong K.H., Matsumura T., Shimada-Niwa Y., Niwa R., Kang S. (2020). The Drosophila Individual Activity Monitoring and Detection System (DIAMonDS). Elife.

[33] Seong K.H., Kang S. (2022). Noncanonical function of the *Sex lethal* gene controls the protogyny phenotype in *Drosophila melanogaster*. Sci. Rep..

[34] Degen T., Hovestadt T., Mitesser O., Hölker F. (2015). High female survival promotes evolution of protogyny and sexual conflict. PLoS One.

[35] Bainbridge S.P., Bownes M. (1981). Staging the metamorphosis of *Drosophila melanogaster*. J. Embryol. Exp. Morphol..

[36] Belgacem Y.H., Martin J.R. (2002). Neuroendocrine control of a sexually dimorphic behavior by a few neurons of the pars intercerebralis in *Drosophila*. Proc. Natl. Acad. Sci. USA.

[37] Isaac R.E., Li C., Leedale A.E., Shirras A.D. (2010). *Drosophila* male sex peptide inhibits siesta sleep and promotes locomotor activity in the post-mated female. Proc. Biol. Sci..

[38] Ribeiro I.M.A., Drews M., Bahl A., Machacek C., Borst A., Dickson B.J. (2018). Visual Projection Neurons Mediating Directed Courtship in *Drosophila*. Cell.

[39] Konopka R.J., Benzer S. (1971). Clock mutants of *Drosophila melanogaster*. Proc. Natl. Acad. Sci. USA.

[40] Myers E.M., Yu J., Sehgal A. (2003). Circadian control of eclosion: Interaction between a central and peripheral clock in *Drosophila melanogaster*. Curr. Biol..

[41] Skopik S.D., Pittendrigh C.S. (1967). Circadian systems, II. The oscillation in the individual *Drosophila* pupa; its independence of developmental stage. Proc. Natl. Acad. Sci. USA.

[42] Sakai T., Ishida N. (2001). Circadian rhythms of female mating activity governed by clock genes in *Drosophila*. Proc. Natl. Acad. Sci. USA.

[43] Sakata K., Kawasaki H., Suzuki T., Ito K., Negishi O., Tsuno T., Tsuno H., Yamazaki Y., Ishida N. (2015). Inositols affect the mating circadian rhythm of *Drosophila melanogaster*. Front. Pharmacol..

[44] Hudry B., de Goeij E., Mineo A., Gaspar P., Hadjieconomou D., Studd C., Mokochinski J.B., Kramer H.B., Plaçais P.Y., Preat T., Miguel-Aliaga I. (2019). Sex Differences in Intestinal Carbohydrate Metabolism Promote Food Intake and Sperm Maturation. Cell.

[45] Churchill E.R., Dytham C., Thom M.D.F. (2019). Differing effects of age and starvation on reproductive performance in *Drosophila melanogaster*. Sci. Rep..

[46] Schultzhaus J.N., Carney G.E. (2017). Dietary protein content alters both male and female contributions to *Drosophila melanogaster* female post-mating response traits. J. Insect Physiol..

[47] Fricke C., Bretman A., Chapman T. (2008). Adult male nutrition and reproductive success in *Drosophila melanogaster*. Evolution.

[48] Klepsatel P., Knoblochová D., Girish T.N., Dircksen H., Gáliková M. (2020). The influence of developmental diet on reproduction and metabolism in *Drosophila*. BMC Evol. Biol..

[49] Schultzhaus J.N., Bennett C.J., Iftikhar H., Yew J.Y., Mallett J., Carney G.E. (2018). High fat diet alters *Drosophila melanogaster* sexual behavior and traits: decreased attractiveness and changes in pheromone profiles. Sci. Rep..

[50] Lebreton S., Carlsson M.A., Witzgall P. (2017). Insulin Signaling in the Peripheral and Central Nervous System Regulates Female Sexual Receptivity during Starvation in *Drosophila*. Front. Physiol..

[51] Kuo T.H., Fedina T.Y., Hansen I., Dreisewerd K., Dierick H.A., Yew J.Y., Pletcher S.D. (2012). Insulin signaling mediates sexual attractiveness in *Drosophila*. PLoS Genet..

[52] Edgecomb R.S., Harth C.E., Schneiderman A.M. (1994). Regulation of feeding behavior in adult *Drosophila melanogaster* varies with feeding regime and nutritional state. J. Exp. Biol..

[53] Ja W.W., Carvalho G.B., Mak E.M., de la Rosa N.N., Fang A.Y., Liong J.C., Brummel T., Benzer S. (2007). Prandiology of *Drosophila* and the CAFE assay. Proc. Natl. Acad. Sci. USA.

[54] Skorupa D.A., Dervisefendic A., Zwiener J., Pletcher S.D. (2008). Dietary composition specifies consumption, obesity, and lifespan in *Drosophila melanogaster*. Aging Cell.

[55] Seeley C., Dukas R. (2011). Teneral matings in fruit flies: male coercion and female response. Anim. Behav..

[56] Rings A., Goodwin S.F. (2019). To court or not to court - a multimodal sensory decision in *Drosophila* males. Curr. Opin. Insect Sci..

[57] Clowney E.J., Iguchi S., Bussell J.J., Scheer E., Ruta V. (2015). Multimodal Chemosensory Circuits Controlling Male Courtship in *Drosophila*. Neuron.

[58] Kohatsu S., Yamamoto D. (2015). Visually induced initiation of *Drosophila* innate courtship-like following pursuit is mediated by central excitatory state. Nat. Commun..

[59] Kimura K.I., Hachiya T., Koganezawa M., Tazawa T., Yamamoto D. (2008). Fruitless and doublesex coordinate to generate male-specific neurons that can initiate courtship. Neuron.

[60] Zhang S.X., Rogulja D., Crickmore M.A. (2016). Dopaminergic Circuitry Underlying Mating Drive. Neuron.

[61] Zhang S.X., Miner L.E., Boutros C.L., Rogulja D., Crickmore M.A. (2018). Motivation, Perception, and Chance Converge to Make a Binary Decision. Neuron.

[62] Takayanagi-Kiya S., Kiya T. (2019). Activity-dependent visualization and control of neural circuits for courtship behavior in the fly *Drosophila melanogaster*. Proc. Natl. Acad. Sci. USA.

[63] Gatti S., Ferveur J.F., Martin J.R. (2000). Genetic identification of neurons controlling a sexually dimorphic behaviour. Curr. Biol..

[64] Stromnaes O., Kvelland I. (1962). Sexual Activity of *Drosophila Melanogaster* Males. Hereditas.

[65] Koppik M., Specker J.H., Lindenbaum I., Fricke C. (2018). Physiological Maturation Lags Behind Behavioral Maturation in Newly Eclosed *Drosophila melanogaster* Males. Yale J. Biol. Med..

[66] Ruhmann H., Wensing K.U., Neuhalfen N., Specker J.H., Fricke C. (2016). Early reproductive success in *Drosophila* males is dependent on maturity of the accessory gland. Behav. Ecol..

[67] Arnes M., Alaniz M.E., Karam C.S., Cho J.D., Lopez G., Javitch J.A., Santa-Maria I. (2019). Role of Tau Protein in Remodeling of Circadian Neuronal Circuits and Sleep. Front. Aging Neurosci..

[68] Das A., Holmes T.C., Sheeba V. (2015). dTRPA1 Modulates Afternoon Peak of Activity of Fruit Flies *Drosophila melanogaster*. PLoS One.

[69] Kistenpfennig C., Nakayama M., Nihara R., Tomioka K., Helfrich-Förster C., Yoshii T. (2018). A Tug-of-War between Cryptochrome and the Visual System Allows the Adaptation of Evening Activity to Long Photoperiods in *Drosophila melanogaster*. J. Biol. Rhythms.

[70] Ratliff E.P., Mauntz R.E., Kotzebue R.W., Gonzalez A., Achal M., Barekat A., Finley K.A., Sparhawk J.M., Robinson J.E., Herr D.R. (2015). Aging and Autophagic Function Influences the Progressive Decline of Adult *Drosophila* Behaviors. PLoS One.

[71] Tomita J., Ueno T., Mitsuyoshi M., Kume S., Kume K. (2015). The NMDA Receptor Promotes Sleep in the Fruit Fly, *Drosophila melanogaster*. PLoS One.

[72] Ferreiro M.J., Pérez C., Marchesano M., Ruiz S., Caputi A., Aguilera P., Barrio R., Cantera R. (2017). *Drosophila melanogaster White* Mutant *w*^1118^ Undergo Retinal Degeneration. Front. Neurosci..

[73] Cosens D., Briscoe D. (1972). A switch phenomenon in the compound eye of the white-eyed mutant of *Drosophila melanogaster*. J. Insect Physiol..

[74] Kalmus H. (1943). The optomotor responses of some eye mutants of *Drosophila*. J. Genet..

[75] Xiao C., Qiu S. (2021). Frequency-specific modification of locomotor components by the *white* gene in *Drosophila melanogaster* adult flies. Genes Brain Behav..

[76] Sitaraman D., Zars M., Laferriere H., Chen Y.C., Sable-Smith A., Kitamoto T., Rottinghaus G.E., Zars T. (2008). Serotonin is necessary for place memory in *Drosophila*. Proc. Natl. Acad. Sci. USA.

[77] Myers J.L., Porter M., Narwold N., Bhat K., Dauwalder B., Roman G. (2021). Mutants of the white ABCG Transporter in *Drosophila melanogaster* Have Deficient Olfactory Learning and Cholesterol Homeostasis. Int. J. Mol. Sci..

[78] Krstic D., Boll W., Noll M. (2013). Influence of the *White* locus on the courtship behavior of *Drosophila* males. PLoS One.

[79] Xiao C., Qiu S., Robertson R.M. (2017). The *white* gene controls copulation success in *Drosophila melanogaster*. Sci. Rep..

[80] Seong K.H., Ly N.H., Katou Y., Yokota N., Nakato R., Murakami S., Hirayama A., Fukuda S., Kang S., Soga T. (2020). Paternal restraint stress affects offspring metabolism via ATF-2 dependent mechanisms in *Drosophila melanogaster* germ cells. Commun. Biol..

