# Supplementary material for: Road to sexual maturity: Behavioral event schedule from eclosion to first mating in each sex of Drosophila melanogaster
Source: iScience. 2023 Jul 28;26(9):107502. doi: 10.1016/j.isci.2023.107502 (PMC10448111; doi:10.1016/j.isci.2023.107502)
Supplement: Documents S1. Figures S1–S9 and Tables S1–S9 [file mmc1.pdf]

iScience, Volume *26*

Supplemental information

Road to sexual maturity: Behavioral event schedule from eclosion to first mating in each sex of *Drosophila melanogaster*

Ki-Hyeon Seong, Tadashi Uemura, and Siu Kang

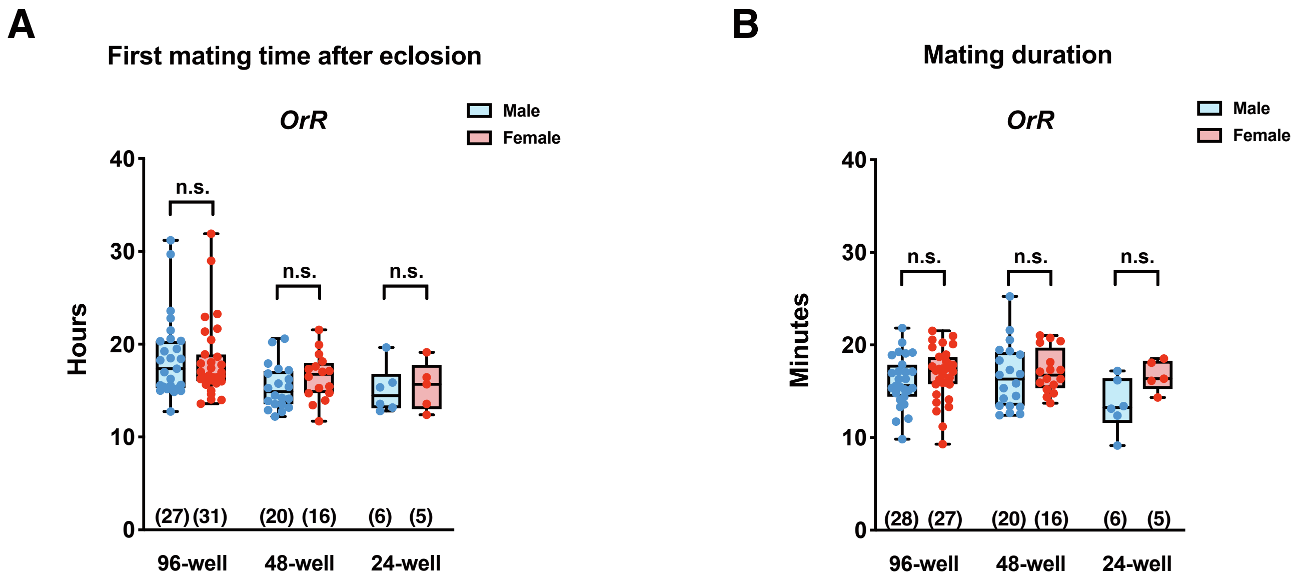

Figure S1. The duration until first mating from eclosion in *D. melanogaster* is not affected by plate well size and partner age, related to Figure 1. (A,B) Box plots illustrating the duration of first mating post eclosion (A) and mating duration (B), respectively, in 96-, 48-, and 24-well microplates following eclosion. The whiskers indicate minima and maxima (n.s., no significant difference; Student' s unpaired t-test). The number of flies analyzed is indicated in parentheses in all graphs.

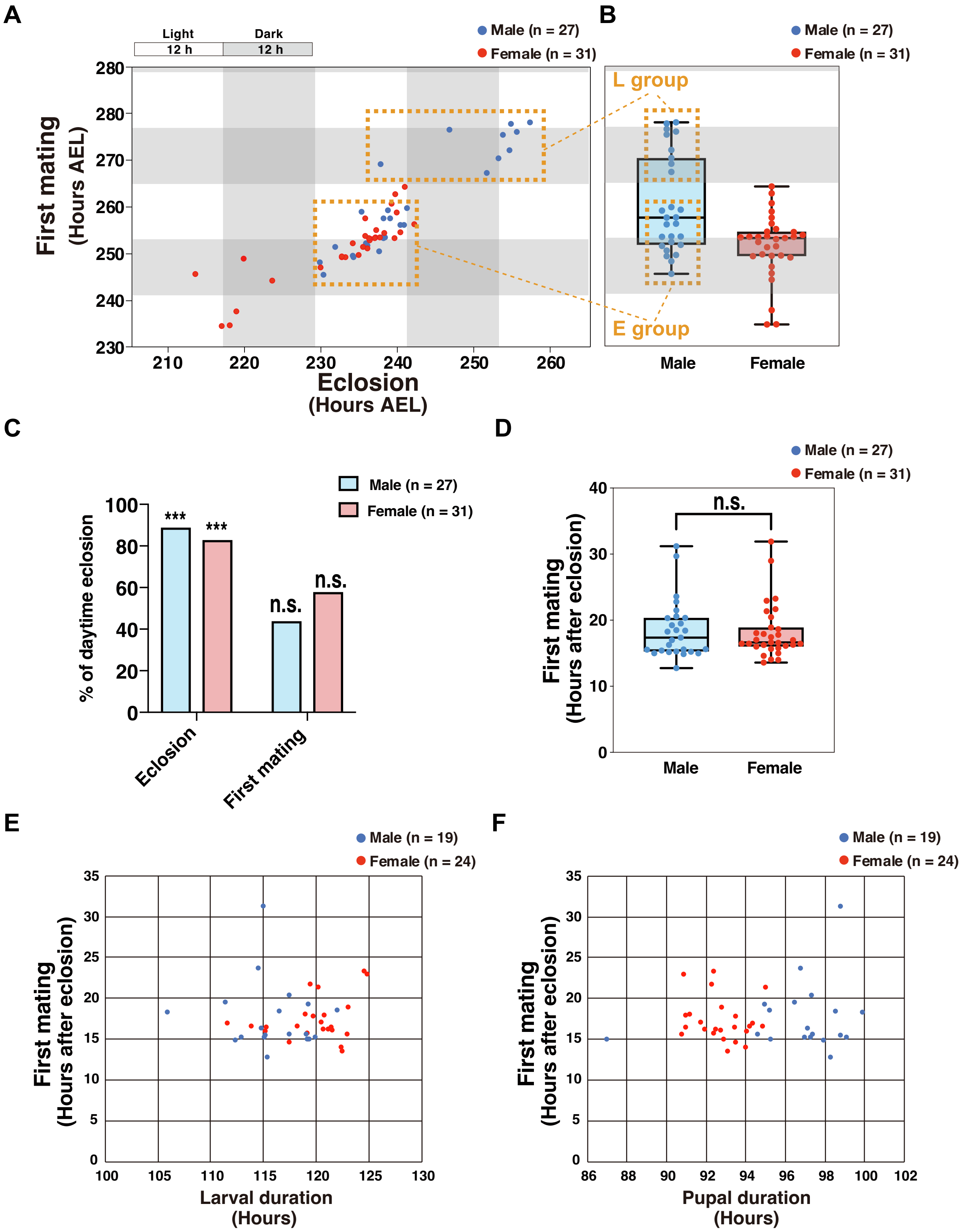

Figure S2. The time of the first mating in *D. melanogaster* is determined as time elapsed after eclosion, related to Figure 2. (A-F) Utilizing the DIAMonDS methodology, we quantified the duration from egg laying to first mating and various life-cycle milestones, such as pupation and emergence. (See "METHOD DETAILS") (A) Scatter plots depict the correlation between the eclosion and first mating times from egg laying, with the X- and Y-axes representing the absolute times of eclosion and first mating (hours after egg laying; hours AEL), respectively. The white and gray color bands within the graph denote the light (L) and dark (D) periods on the X- and Y-axes. (B) Box plots of the first mating time from egg laying in both sexes (data is derived from the same dataset as in (A). Whiskers indicate minima and maxima (n.s., no significant difference; Student' s unpaired t-test). (C) Bar graphs depict the percent preference for the light period of eclosion and first mating (***, $p < 0.0001$; n.s., no significant difference; Binomial test). (D) Box plots of first mating time after egg laying. Whiskers indicate minima and maxima (n.s., no significant difference; Student' s unpaired t-test). (E) Scatter plots of the duration of the larval stage and first mating time after eclosion. (F) Scatter plots of the period of the pupal stage and first mating time after eclosion. The number of flies analyzed is indicated in parentheses on all graphs.

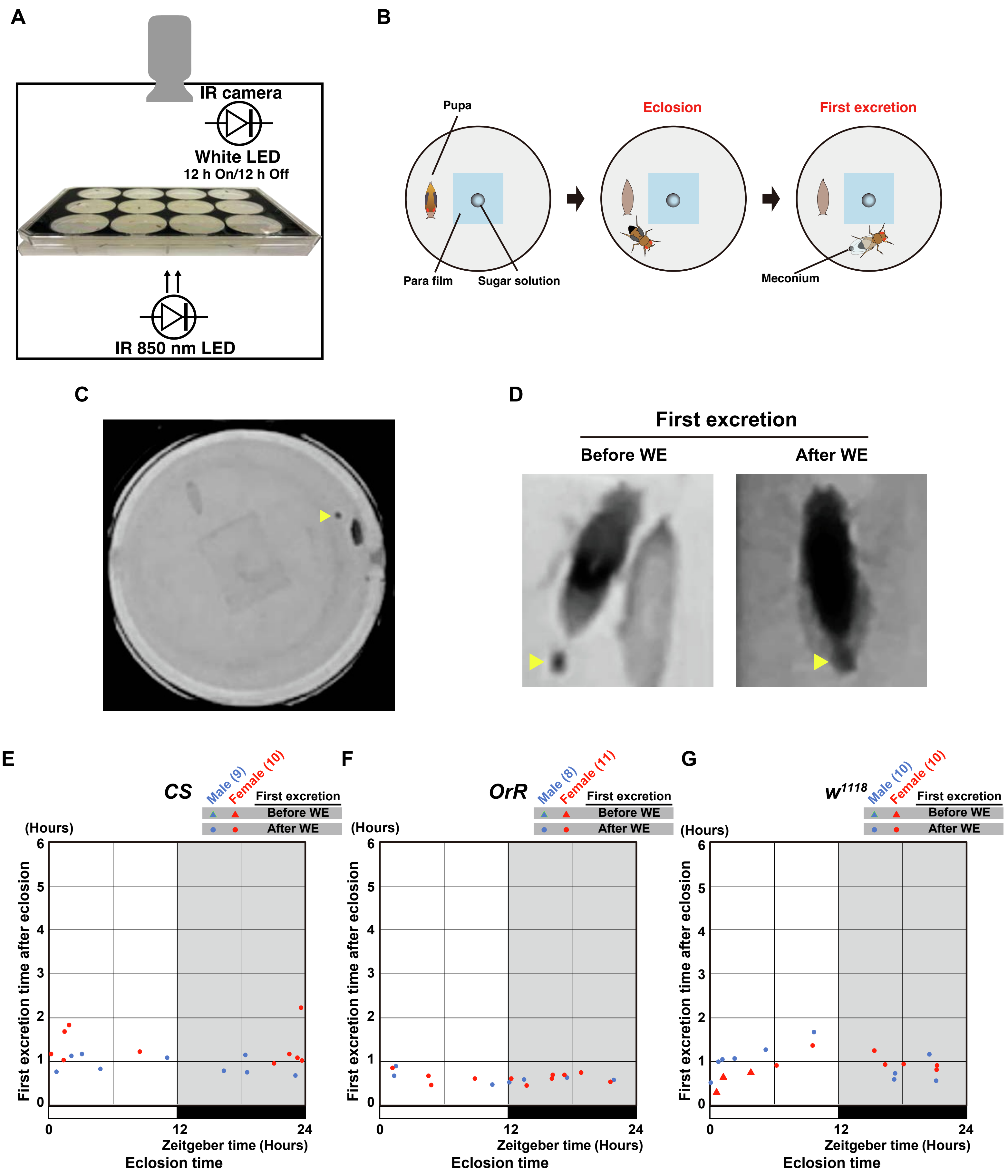

Figure S3. Detection of first excretion in *D. melanogaster*, related to Figure 3. (A, B) Schematic representation of the method used for detecting the time of eclosion and first excretion. A black pupa was placed in each well of a 12-well microplate and observed until first excretion event (A). In each well of a 12-well microplate, a droplet of sugar solution put on the parafilm (B). (C) Schematic images of the first excretion. Arrowhead indicates the meconium. (D) Images of the first excretion before and after wing expansion (WE). Arrowhead indicates a meconium. Scatter plots of the eclosion time and the first excretion time after eclosion in the (E) *CS*, (F) *OrR*, and (G) *w^{1118}* strains. Number of flies analyzed is indicated in parentheses in all graphs.

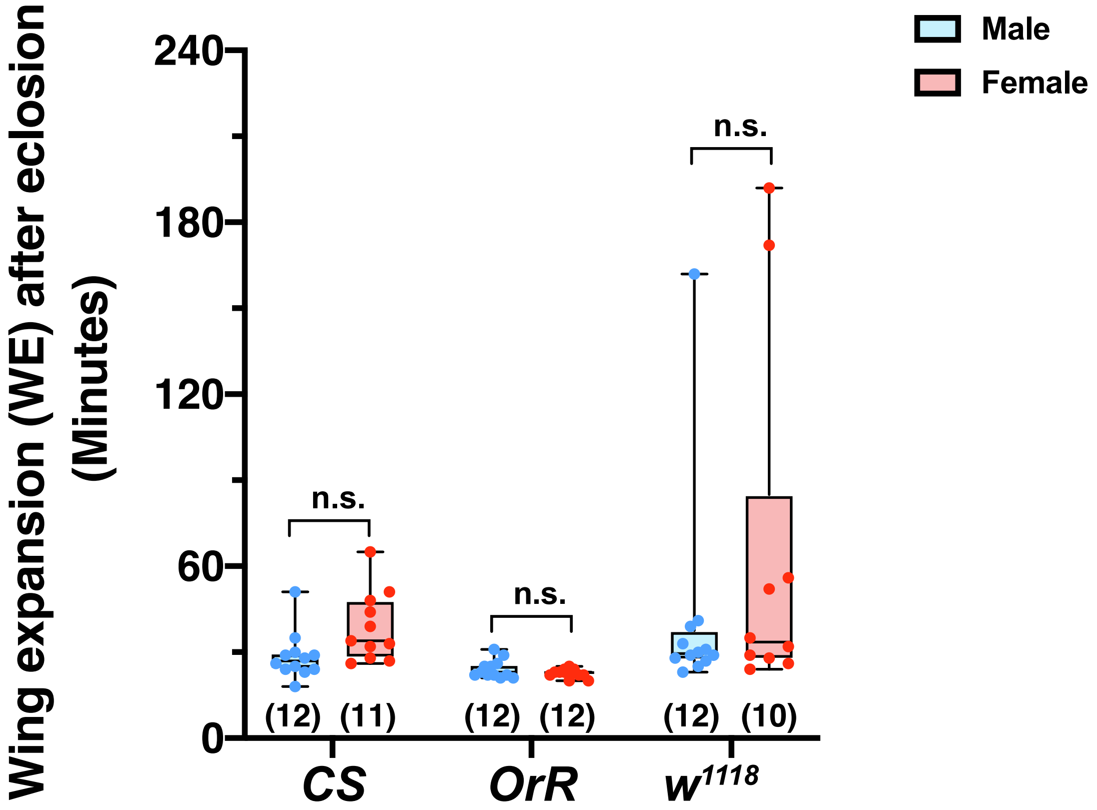

Figure S4. Wing expansion timing after eclosion, related to Figure 3. Box plots of wing expansion after eclosion in each sex of the *CS*, *OrR*, and w^{1118} strains. Whiskers indicate minima and maxima (n.s., no significant difference; Student' s unpaired t-test). Number of flies analyzed is indicated in parentheses in all graphs.

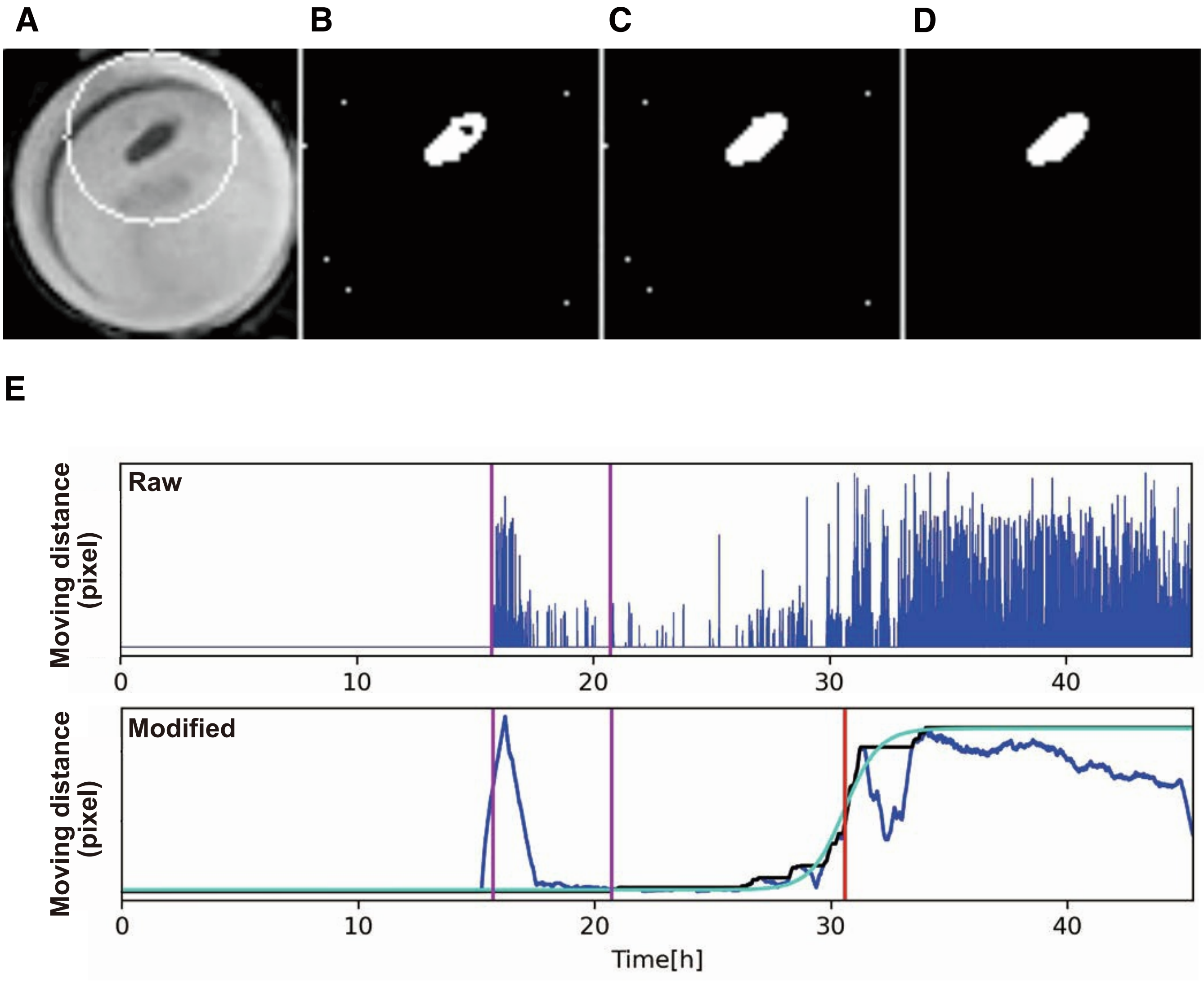

Figure S5. Example of image processing and time series analysis for estimation of activity after eclosion, related to Figure 4A. (A) A raw image of a fly in a well extracted as a square shape. A white circle indicates a target fly. (B) Detected binary image of the fly body as a foreground extraction. Closing (C) and opening (D) operation after the extraction for object filling and noise reduction. (E) An example of activity elevation. (Upper panel) The blue line indicates the time series of instantaneous displacement calculated between pre- and post-images. Two magenta lines correspond to the eclosion onset manually detected, and the eclosion offset is automatically defined as a fixed value after the onset. (Lower panel) The blue line indicates a smoothing of raw displacement in the upper panel. The black line corresponds modified signal described in the main text. The cyan line is a sigmoid curve-fitting on the modified signal. The red vertical line indicates a time point of activity elevation defined as a activity phase transition time (APTT) point of the sigmoid function.

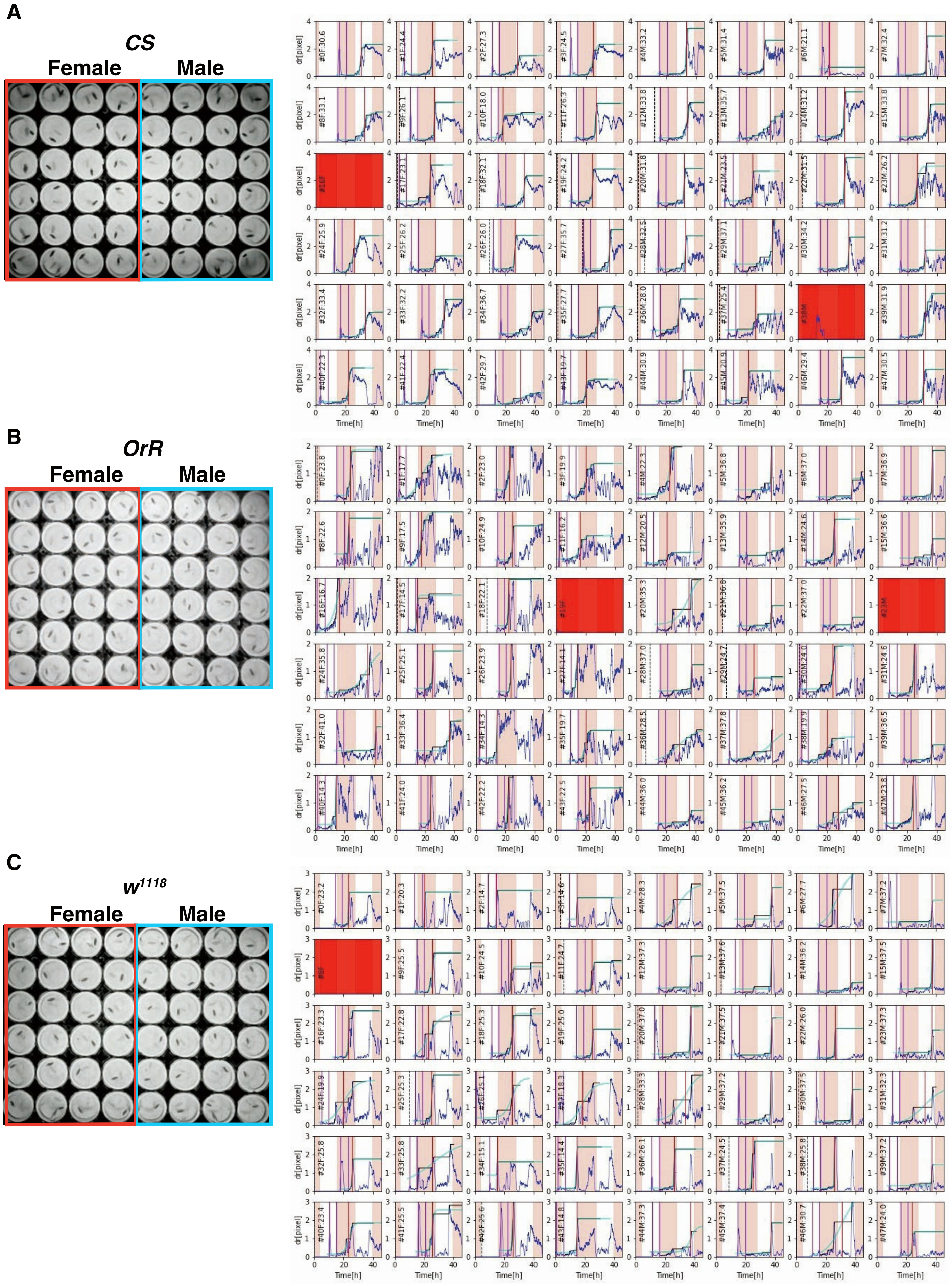

Figure S6. Individual variation in phase transition of locomotor activity just after eclosion in a 12:12 hour light-dark (LD) cycle condition, related to Figure 4D. (A) *Canton-S* (*CS*), (B) *Oregon-R* (*OrR*), and (C) *white*^{1118} (*w*^{1118}) strains. Blue line indicates smoothing activity obtained by instantaneous displacement of gravity center of Drosophila (see "Method details"). Two vertical purple lines describe eclosion. Cyan line was time course of tentative maximum activity and black line was sigmoid fitting on it. Orange shaded area indicates the light phase of LD cycle. Red shaded panel is the died fly without eclosion. This figure is corresponding to Figure 4B and D.

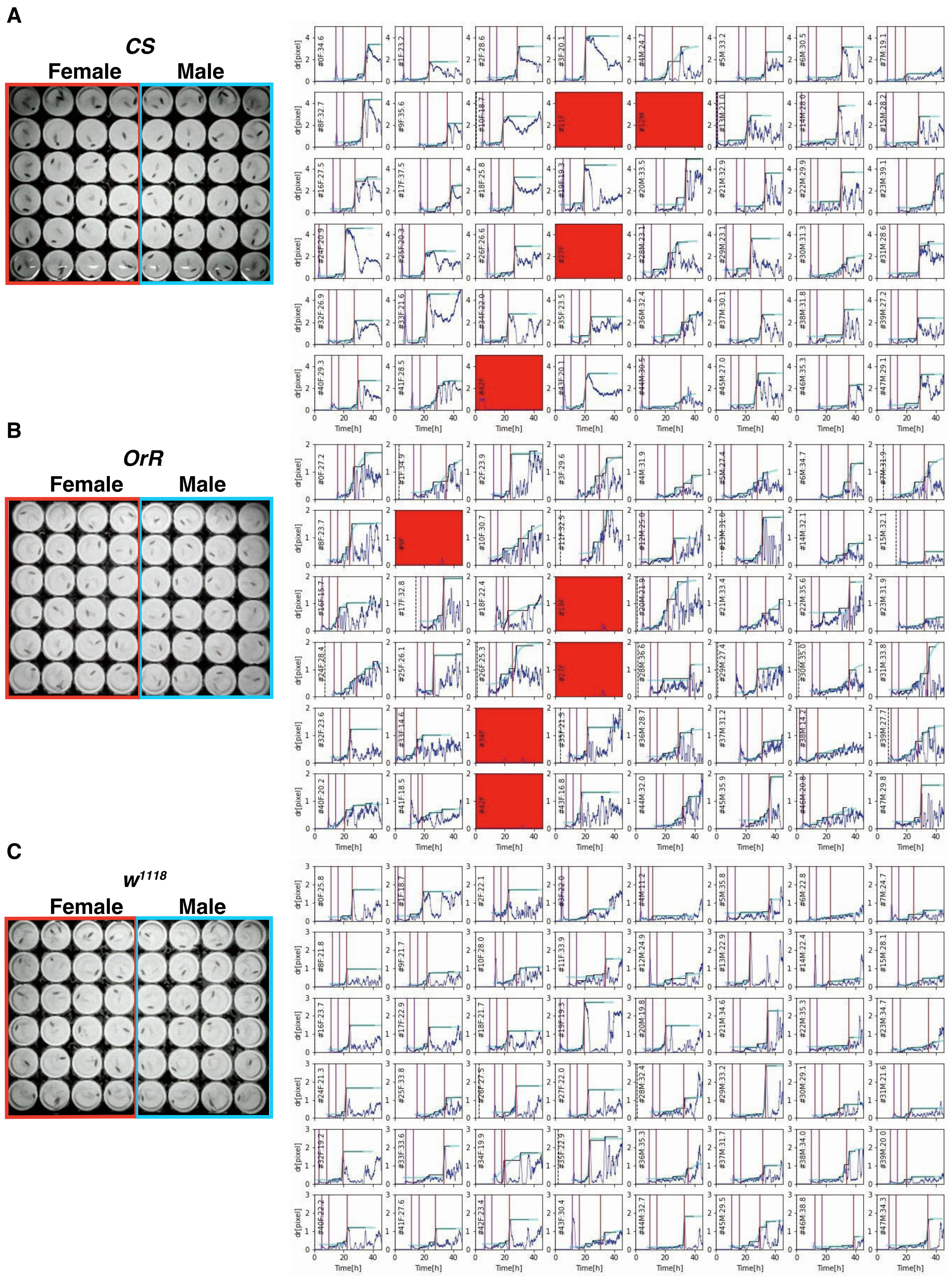

Figure S7. Individual variation in phase transition of locomotor activity just after eclosion in a dark-dark (DD) cycle condition, related to Figure 4E. (A) *Canton-S* (*CS*), (B) *Oregon-R* (*OrR*), and (C) *white*^{1118} (*w*^{1118}) strains. Blue line indicates smoothing activity obtained by instantaneous displacement of gravity center of Drosophila (see "Method details"). Two vertical purple lines describe eclosion. Cyan line was time course of tentative maximum activity and black line was sigmoid fitting on it. Red shaded panel is the died fly without eclosion. This figure is corresponding to Figure 4C and E.

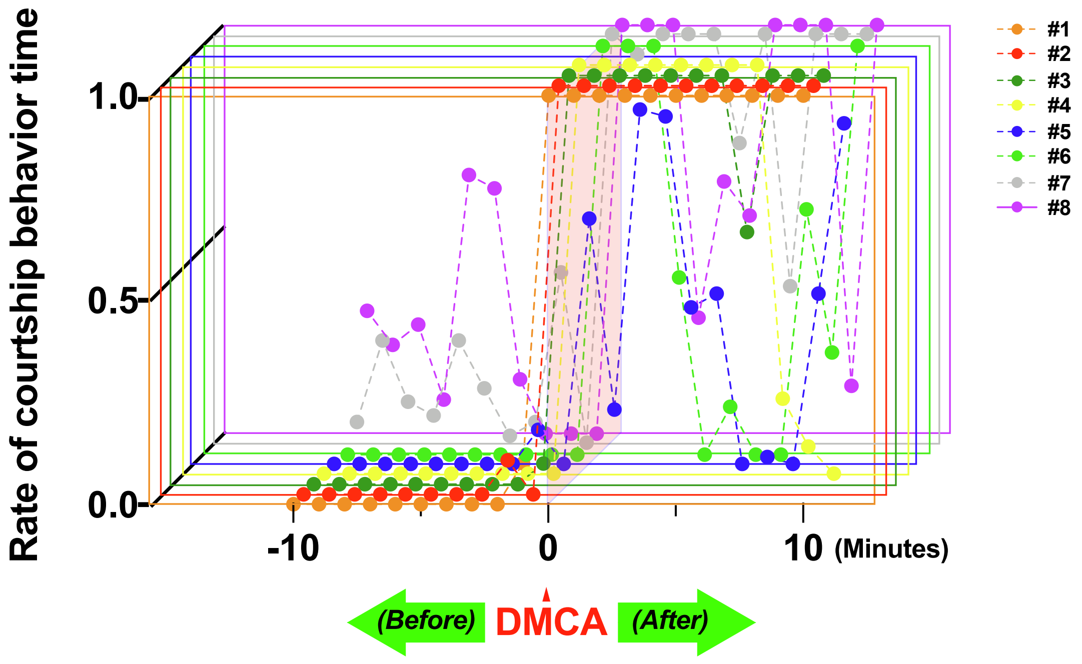

Figure S8. Individual variation in courtship activity rate for ±10 min before/after the DMCA (drastic male courtship arousal) point, related to Figure 5E. Each layer shows the changes in courtship activity rate before and after DMCA for each of the males (#1- #8) used in Figure 5E.

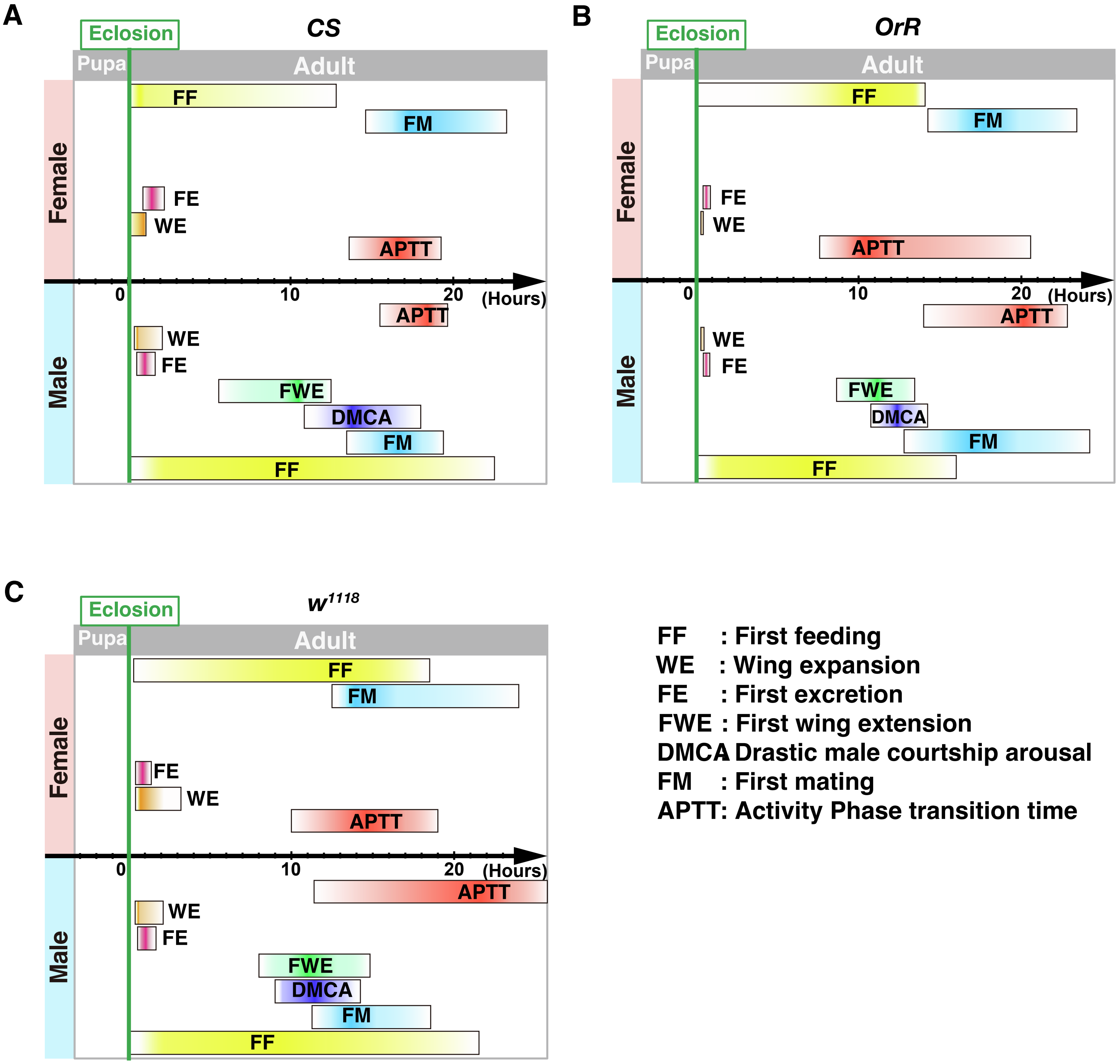

Figure S9. Schematic schedule of behavioral events during sexual maturation from the eclosion to the first mating in each sex of individual *CS* (A), *OrR* (B), and w^{1118} (C) strains, related to Figure 6. The boxes represent the range of minima and maxima, and the darkest color and its gradients indicate the mean and distribution of each event. (yellow) FF, First feeding; (gold) WE, Wing expansion; (magenta) FE, first excretion; (green) FWE, first wing extension; (blue) DMCA, drastic male courtship arousal; (light blue) FM, first mating; and (red) APTT, activity phase transition time.

Table S1. Two-way ANOVA of first mating time post eclosion, related to Figure 1D-1F.

	ss	df	F-value	p-value
Sex	3.7516	1	0.2672	0.6060
Strain	255.0818	2	9.0849	0.0002
Sex x Strain	42.9453	2	1.5295	0.2200
Residual	2091.7703	149	NaN	NaN

Table S2.Two-way ANOVA of mating duration, related to Figure 1G-1I.

	ss	df	F-value	p-value
Sex	81.1230	1	8.6093	0.0039
Strain	635.8497	2	33.7402	8.E-13
Sex x Strain	123.5633	2	6.5567	0.0019
Residual	1403.9891	149	NaN	NaN

Table S3.Two-way ANOVA of first mating time after eclosion in (CS, Food), related to Figure 5G.

	ss	df	F-value	p-value
Sex	13.8706	1	1.4056	0.2399
Food	78.3876	1	7.9437	0.0063
Sex x Food	20.5208	1	2.0795	0.1539
Residual	671.0187	68	NaN	NaN

Table S4. Two-way ANOVA of first mating time after eclosion (OrR, Food), related to Figure 5H.

	ss	df	F-value	p-value
Sex	22.6581	1	1.6889	0.1984
Food	7.9553	1	0.5930	0.4441
Sex x Food	20.4052	1	1.5210	0.2220
Residual	858.5978	64	NaN	NaN

Table S5. Two-way ANOVA of first mating time after eclosion (WT, Food), related to Figure 5I.

	ss	df	F-value	p-value
Sex	25.2572	1	2.1385	0.1472
Food	24.6053	1	2.0833	0.1525
Sex x Food	8.0721	1	0.6835	0.4106
Residual	1039.3397	88	NaN	NaN

Table S6. Two-way ANOVA of moving distance after APTT (LD), related to Figure 4B.

	ss	df	F-value	p-value
Sex	13.7782	1	41.9759	2.E-09
Strain	71.7295	2	109.2636	8.E-28
Sex x Strain	1.5409	2	2.3472	0.1000
Residual	39.7171	121	NaN	NaN

Table S7. Two-way ANOVA of moving distance from APTT (DD), related to Figure 4C.

	ss	df	F-value	p-value
Sex	7.9020	1	20.6266	1.E-05
Strain	140.5757	2	183.4718	8.E-37
Sex x Strain	1.6501	2	2.1536	0.1206
Residual	44.8226	117	NaN	NaN

Table S8. Two-way ANOVA of APTT from eclosion (LD), related to Figure 4D.

	ss	df	F-value	p-value
Sex	1258.0300	1	93.0785	1.E-16
Strain	68.4641	2	2.5327	0.0836
Sex x Strain	419.9136	2	15.5342	1.E-06
Residual	1635.4117	121	NaN	NaN

Table S9. Two-way ANOVA of APTT from eclosion (DD), related to Figure 4E.

	ss	df	F-value	p-value
Sex	645.7530	1	52.0975	6.E-11
Strain	113.9810	2	4.5978	0.0120
Sex x Strain	24.5812	2	0.9916	0.3741
Residual	1450.2242	117	NaN	NaN

ss = sum of squares. df = degrees of freedom.
